# Supplementary material for: Are carbon emissions trading and green financial instruments synergistic? -Comprehensive quantitative research based on content analysis
Source: PLoS One. 2024 Mar 7;19(3):e0298601. doi: 10.1371/journal.pone.0298601 (PMC10919601; doi:10.1371/journal.pone.0298601)
Supplement: S1 Data — (ZIP) [file pone.0298601.s001.zip › Data/地级市Z_F工作报告.zip!/╕ú╜¿/─■╡┬╩╨/└┤╫╘╣1⁄2╓┌║┼í╛┬φ┐╦╩2╛▌═°í┐_2019.docx]

各位代表：

　　现在，我代表宁德市人民政府，向大会报告政府工作，请予审议，并请市政协委员提出意见。

　　一、2018年工作回顾

　　2018年是全面贯彻落实党的十九大精神开局之年，是改革开放40周年。我市各级政府坚持以习近平新时代中国特色社会主义思想为指导，认真贯彻党的十九大和十九届二中、三中全会精神，坚决落实省委、省政府和市委决策部署，坚持稳中求进工作总基调，坚持新发展理念，坚持高质量发展落实赶超，全面实施“一二三”发展战略，扎实推进稳增长、促改革、调结构、惠民生、防风险工作，经济社会发展取得新成效，较好地完成了市四届人大二次会议确定的各项目标任务。全市生产总值1942.8亿元、增长8.1%；一般公共预算总收入200.77亿元、增长11.7%，地方一般公共预算收入120.42亿元、增长9.1%，增速均居全省首位；固定资产投资增长9.6%；出口总值216.69亿元、增长6.9%；实际利用外资1.2亿元、增长91.4%（新口径）；社会消费品零售总额611.11亿元、增长8.1%；居民消费价格总水平上涨1.2%；城镇居民人均可支配收入32921元、增长7.9%；农村居民人均可支配收入16147元、增长9.7%，增速居全省首位；城镇登记失业率2.72%。

　　一年来的主要工作和成效是：

　　（一）抓重点、出实招，三大攻坚战初战告捷。金融风险加快化解。政司银企联动协作持续深化，处置不良贷款67.9亿元，不良贷款率降至1.68%，降幅居全省首位，创2013年以来最低。扎实推进国家普惠金融改革试验区创建工作，建成信贷“黑、白、灰”名单信息库和中小微企业信用信息交换与共享平台，商业银行贷款审批权限逐步下放，全市金融机构本外币存款余额1843.03亿元、增长20.6%，增速居全省首位，贷款余额1744.29亿元、增长10.6%，增速居全省第三位。精准脱贫成效显著。现行国定标准贫困人口全部脱贫，省定标准贫困人口减少到193人，贫困发生率下降至万分之一以下。霞浦率先实现脱贫“摘帽”。完成造福工程搬迁5849人。发放小额信贷扶持资金6.2亿元，投入产业扶贫资金1.35亿元，实施产业、就业扶贫项目2.48万个。新增健康扶贫补充保险，扶贫救助保险实现全覆盖。扶贫开发主题展示馆建成投用。寿宁下党荣获全国脱贫攻坚组织创新奖。扶贫开发“宁德模式”进一步丰富拓展。污染防治强力推进。14项中央环保督察年度整改任务顺利完成。海上养殖综合整治全面展开，累计清退渔排12.2万口、藻类2.4万亩，升级改造渔排7.5万口、藻类1.8万亩。县级污水处理厂完成提标改造。河湖长制实现全覆盖。劣五类小流域和中心城区黑臭水体基本消除，主要流域水质优良比例达100%。火电、冶金等重点行业完成大气污染物减排治理，合成革集中区全面实现集中供热。农用地土壤污染状况详查全面完成。市危废综合处置中心和青拓、联德废渣综合利用项目建成投用，福鼎生活垃圾焚烧发电厂基本建成。治理水土流失22.2万亩。植树造林11.62万亩，实现省级森林城市全覆盖。柘荣获评国家生态文明建设示范县。

　　（二）抓龙头、建集群，产业发展提质增效。工业主导地位凸显。实现规上工业增加值增长9.2%，增速居全省首位。工业用电量增长14.9%，增速居全省第二位。全市研发投入增长22%，新增省级以上高新技术企业30家，高新技术产业增加值增长16.2%、占规上工业增加值42.1%。全市产值超百亿元企业达7家，青拓集团成为全省首家年产值超千亿民营企业。“宁德制造”快速崛起，锂电新能源、不锈钢新材料、铜材料三大产业增加值增长20.4%，对全市规上工业增加值贡献率达107.5%。宁德时代获批国家高新技术产业标准化试点和第三批制造业单项冠军企业，牵头组建电化学储能技术国家工程研究中心，主导制定电动汽车安全全球技术法规，成为国内第三家、省内首家在A股上市的“独角兽”企业。时代新能源三期、新能源科技三期开工建设。青拓特钢、高速线材型材、上克不锈钢等项目建成投产，不锈钢新材料产业实现产值1046.97亿元，成为我市首个千亿产业集群。中铝40万吨铜冶炼项目仅用17个月实现投产，刷新了项目建设“宁德速度”。三祥新材1万吨氧化锆、中信重工特种机器人、思客琦一期等项目建成投产。时代电机成为吉利汽车优秀供应商。广生堂新一代抗乙肝病毒药品在全省率先通过仿制药质量与疗效一致性评价。出台支持建筑业发展壮大9条措施，新增建筑业企业156家，产值增长13.1%。现代服务业加速培育。实现服务业增加值678.85亿元、增长9.7%。全域旅游加快发展，旅游总收入343亿元、增长35%。第八届宁德世界地质公园文化旅游节成功举办，宁德世界地质公园顺利完成联合国教科文组织第二次中期评估。首批26个旅游小镇加快建设。蕉城霍童、寿宁西浦入选国家水利风景区。周宁鲤鱼溪成为全国首个国家级鱼文化主题公园。商贸流通持续繁荣，新增限上贸易企业178家。电子商务交易额678亿元、增长26%，寿宁、柘荣入选国家级电子商务进农村综合示范县。港口物流初具规模，吞吐量突破3000万吨、增长15.5%，其中集装箱吞吐量9.6万标箱、增长12%，增速均居全省第二位。特色现代农业稳步发展。实现农林牧渔业增加值300.26亿元、增长4.3%。粮食安全责任制有效落实，建成高标准农田24.5万亩。八大特色农业产业加快转型发展。福鼎白茶产区和宁德大黄鱼产区入选中国特色农产品优势区。食用菌工厂化、标准化栽培加快推广应用。柘荣太子参规模化产业基地建成投产。新认定农业产业化国家重点龙头企业2家。新增“三品一标”农产品49个。农村土地承包经营确权登记颁证率95.9%，“两权”抵押贷款余额18.3亿元。选认省市县科技特派员423名，实现乡镇全覆盖。福鼎入选全国农村综合性改革试点试验区。古田列入全国农村一二三产业融合发展先导区创建名单。

　　（三）抓招商、扩投资，项目建设加快推进。超额完成千亿招商任务，签约合同项目349个、总投资1234.5亿元，落地率73.3%、开工率58.2%。新增“五个一批”项目1456个，其中谋划426个、签约406个、开工409个、投产172个、增资43个，工作经验在全省推进会上作交流发言。蕉城荣获全省第三季度“五个一批”项目正向激励综合考评第一名。成功引进青美新能源材料、格林美循环经济产业园、时代一汽、鼎盛精品钢、奥展不锈钢产业示范园、上汽创投、振华汽车部件、沪光线束等一批大项目好项目。16个省赶超发展在建重大项目完成投资102亿元，完成年度计划的113.2%。300个市在建重点项目完成投资640亿元，开工120个、竣工70个。上汽宁德基地项目8个月完成投资126亿元、占总投资的63%，主机厂完成主体框架建设，再次刷新了项目建设“宁德速度”。上汽铁路专用线、沈海高速三屿互通、七都溪特大桥等上汽宁德基地配套项目加快建设，带动基础设施建设水平大跨步提升。衢宁铁路宁德段进入全线铺轨阶段。沈海复线福鼎管阳至柘荣段、福安至蕉城漳湾段和屏古联络线等3条（段）高速建成通车，新增通车里程117公里。霞浦浮鹰岛风电场建成投产，周宁抽水蓄能电站顺利截流蓄水。漳湾作业区10#、湾坞作业区12#13#泊位建成投用。

　　（四）抓基础、强功能，城乡面貌持续改善。中心城区功能不断优化。三都澳新区核心区完成控规及启动区城市设计，宁德一中新校区开工建设。宁德火车站综合客运枢纽站一期建成投用。城区内外连接通道不断完善，天山东路延伸至沈海复线宁德东互通口，富春东路建成通车，南北湖滨路、八一五路等实现“白改黑”。署前路棚改一期完成主体工程。新增公共停车位400个、充电桩500个。南大塘福瑶路至四孔闸段、南港连通渠、大寨溪支流排洪渠完成主体工程。官昌水库下闸蓄水，第三水厂日供水能力提高到10万吨。城区肉联厂建成投用。建成区绿化覆盖率37.9%。重点区域完成夜景亮化工程。荣获全国文明城市提名城市，创建工作全面启动，全市总动员、全民齐参与氛围不断浓厚。新型城镇化建设步伐加快。古田、福安新型城镇化综合试点工作扎实推进。赛岐、太姥山完成“小城市”培育试点三年行动计划。7个省级特色小镇实施创建项目62个、完成投资53亿元。锂电新能源小镇被推荐为全国特色小镇高质量发展典型案例。全市城镇化率提高到56.8%。乡村人居环境大幅提升。铁路沿线和农村人居环境整治三年行动启动实施，完成行政村改厕765个、改水870个、生活垃圾治理632个。新增中国历史文化名镇2个、名村8个、传统村落78个。路长制和乡村道路专管员制度全面落实，完成农村公路建设改造405公里、安全生命防护工程507公里。周宁获评省级“四好农村路”示范县。高速宽带及4G网络实现行政村全覆盖。

　　（五）抓改革、促开放，发展活力有效增强。出台进一步深化改革扩大开放85条措施。机构改革平稳有序，新组建、新调整机构全面完成挂牌。“放管服”改革纵深推进，营商环境持续提升。市级“一趟不用跑”和“最多跑一趟”事项占比从80%提高至93.9%，不动产登记和企业开办时间缩短至5天。实行市级重大项目“模拟审批”，项目前期审批时限压缩一半以上。“证照分离”改革全面铺开。以管资本为主的国有资产监管体制基本理顺。市级一般公共预算资金绩效管理基本实现全覆盖。税制改革深入推进。市属公立医院人事薪酬制度改革扎实推进，在全省率先启动医疗集团医保总额打包付费改革。国家军民融合创新示范区加快创建。出台促进民营企业加快发展和企业家健康成长等一系列措施。“宁智回归”工程启动实施，香港、广东、上海宁德市人才工作站挂牌成立。出口信保扶持力度加大，实体企业出口增长18%。“一带一路”经贸合作取得突破，5家企业通过境外投资备案，完成对外总投资3.03亿美元。宁德时代在美国、日本成立子公司，在德国建设首个海外工厂。智能发电机组、电喷化油器成功拓展非洲、中东、美国等海外市场。与台港澳交流合作持续深化，第九届海峡两岸电机电器博览会和第十届海峡论坛·陈靖姑文化节成功举办。中国华侨国际文化交流基地落户古田临水宫。

　　（六）抓短板、强弱项，群众获得感不断提升。社会保障力度加大。全市民生支出235.26亿元，占一般公共预算支出78.4%。30件为民办实事项目基本完成。新增城镇就业2.53万人、农村劳动力转移就业4.06万人。蕉城、福鼎、福安农村低保标准从4000元提高到7560元、古田提高到6960元，实现城乡低保标准一体化，其余5个县提高到5100元。基本医保和机关企事业单位退休人员养老金标准稳步提高。全面建立城乡居民养老保险丧葬补助金制度。推行住房公积金按月冲还贷业务。保障性安居工程累计配租配售3.1万套，3.7万户城市居民低收入住房困难户实现应保尽保。公共服务供给加强。实施民生社会事业领域补短板项目472个、完成投资40.3亿元。新增学位2.74万个、医疗床位410张、养老床位1000张。普惠性幼儿园覆盖率75.1%，民办幼儿园进一步规范。“全面改薄”五年规划任务如期超额完成，新增义务教育管理标准化学校58所、省级示范高中2所。宁德职业技术学院通过第二轮高职院校人才培养工作评估。宁德师范学院入选硕士点培育单位，二级医学院实现首批招生。新增省市临床重点专科项目16个，7个县域紧密型医共体投入运营，新建达标一体化村卫生所658个。乡镇敬老院全面达标，居家养老专业化服务实现城乡社区全覆盖，护理型床位占比提高33.9个百分点。文体事业繁荣发展。第十六届省运会、第十届老健会成功举办，获得省运会赛时金牌80枚、居全省第四位，老健会金奖13项、居全省第二位，实现办会参赛“双丰收”，提升了城市建设管理水平和社会文明程度，展现了新时代“宁德形象”。畲族银器制作、柘荣剪纸技艺入选国家传统工艺振兴目录，新增省级文物保护单位20个。畲族文化普查全面完成。福安康厝畲族乡被命名为全国民族团结进步创建示范区。汤养宗诗集《去人间》荣获全省首个鲁迅文学奖诗歌奖。社会大局和谐稳定。启动新一轮“平安宁德”建设，深入开展扫黑除恶专项斗争，严厉打击各类违法犯罪，群众安全感率达98.5%、居全省前列。移风易俗专项治理深入开展，烟花爆竹燃放得到管控。完成“两违”综合治理499.6万平方米。村级组织换届选举工作圆满完成。宗教工作网络和工作机制进一步完善。食品药品监管力度加大。信访工作制度改革深入推进。安全生产形势持续稳定。防灾减灾扎实有效。国防动员、后备力量建设和海防、人防、反走私工作得到加强。工、青、妇、计生协、科协、统计、老区、老龄、残联、移民、人事编制、档案、地方志、气象、防震、外事、侨务、关心下一代等工作都取得新进展。

　　（七）抓作风、严纪律，自身建设得到加强。全面兴起 “大学习”热潮，坚持用习近平新时代中国特色社会主义思想武装头脑、指导实践、推动工作，树牢“四个意识”，坚定“四个自信”，坚决做到“两个维护”。切实履行全面从严治党政治责任，中央巡视反馈和省委全面从严治党主体责任落实情况检查涉及政府系统问题完成整改。坚决执行中央八项规定及其实施细则精神和省、市《实施办法》，党风廉政建设和反腐败工作深入开展。自觉接受人大及其常委会监督并报告工作，依法执行人大及其常委会决定决议，办理市人大代表建议355件。支持政协履行政治协商、民主监督、参政议政职能，办理市政协提案409件。自觉接受监察机关监督和社会监督，审计监督不断加强，机关效能建设持续推进。制定三都澳海域环境保护办法等政府规章和规范性文件56件，规章报备工作受到司法部通报肯定。

　　各位代表，过去一年成绩来之不易，这是中央和省委、省政府正确领导、关心支持的结果，是在市委坚强领导下全市干部群众团结一心、奋力拼搏的结果，是市人大、市政协加强监督、鼎力支持的结果。在此，我代表市人民政府，向全市人民，向人大代表、政协委员、各民主党派、工商联、各人民团体、无党派人士、离退休老同志和社会各界人士，向中央及省属驻宁机构、人民解放军和武警驻宁部队、公安民警，表示衷心的感谢和崇高的敬意！

　　在肯定成绩的同时，我们也清醒地认识到发展中还存在不少困难和问题，主要表现在：山区与沿海发展差距持续拉大，一些地方产业项目不多、发展后劲不足；现代服务业规模偏小，部分中小微企业经营困难加大，减停产增多；自然资源供给和需求的矛盾较为突出，环保任务重、防治压力大；稳定脱贫质量有待提高，发展壮大村级集体经济办法不多；交通堵、停车难等现象突出，教育、医疗、养老等民生社会事业还有不少短板；一些干部担当作为不够，能力与推动高质量发展要求不相适应，发展环境还有待进一步优化等等。对此，我们要高度重视，采取有力措施加以解决。

　　二、扎实做好2019年工作

　　2019年是新中国成立70周年，是坚持高质量发展落实赶超、决胜全面建成小康社会的攻坚年。政府工作的总体要求是：以习近平新时代中国特色社会主义思想为指导，全面贯彻党的十九大和十九届二中、三中全会精神，认真落实中央和省委、市委经济工作会议的部署要求，统筹推进“五位一体”总体布局，协调推进“四个全面”战略布局，坚持稳中求进工作总基调，坚持新发展理念，坚持高质量发展落实赶超，坚持以供给侧结构性改革为主线，坚持深化市场化改革、扩大高水平开放，全面实施“一二三”发展战略，继续打好三大攻坚战，统筹推进稳增长、促改革、调结构、惠民生、防风险工作，进一步稳就业、稳金融、稳外贸、稳外资、稳投资、稳预期，提振市场信心，增强人民群众获得感、幸福感、安全感，保持经济持续健康发展和社会大局稳定，为全面建成小康社会收官打下决定性基础，以优异成绩落实赶超任务、庆祝新中国成立70周年。

　　经济社会发展的主要预期目标是：全市生产总值增长9.5%以上；规上工业增加值增长12%以上；一般公共预算总收入增长6%，地方一般公共预算收入增长5%；固定资产投资增长10%；进出口总值增长6%；实际利用外资增长5%以上；社会消费品零售总额增长10.5%；居民消费价格总水平涨幅控制在3%以内；城镇居民人均可支配收入增长8.5%，农村居民人均可支配收入增长9%；城镇登记失业率控制在4%以内；完成年度各项节能减排任务。

　　实现上述目标，重点抓好七个方面工作：

　　（一）坚持山海统筹，构建协同发展新格局

　　增强湾区经济驱动力。主动融入闽东北协同发展区建设，高起点规划、高标准配套、高效率推进三都澳、福宁湾、沙埕湾三个湾区发展。启动湾区经济发展规划编制，明确总体目标和功能定位。立足做大做强三都澳湾区，重点推动锂电新能源、新能源汽车、不锈钢新材料、铜材料四大主导产业集聚发展，深入实施锂电新能源小镇、不锈钢新材料小镇规划，推动东侨建设高新技术产业园区，支持赛甘下坞组团“腾笼换鸟”、综合开发，推进溪南半岛工业区启动区开发建设。加快建设福宁湾产业发展平台，完善霞浦省级经济开发区、台湾水产品集散中心基础配套，积极布局锂电新能源、新能源汽车等产业配套项目，开工建设时代一汽、罗兰蒂项目，加快弥合闽东沿海工业断裂带。推动沙埕湾开放开发，支持福鼎省级工业园区扩区、龙安工业区升级，主动承接浙南经济辐射，加快建设鼎盛精品钢项目，建成投产国泰电解液项目，促进福建“北大门”快速崛起。

　　增强中心城区承载力。坚持以城促产、产城融合，以一流城市环境承载一流产业发展。开展新一轮城市总体规划修编，加强重点区域城市设计，加快完善城市功能配套。推动温福铁路以东区域连片开发，加快塔南片区“退二进三”，统筹布局环东湖商业服务配套，加强低效、零星用地综合利用。动工建设连城路，打通南北向产城新主轴。加快推进104国道宁古路口至漳湾疏港公路段拓宽工程，建成天德路、金湾路、东井路等交通干道。推动三屿园区纳入市区公交体系。实施提升城市供水水质行动。深化黑臭水体标本兼治，统筹抓好控源截污、污水处理、两岸绿化等重点工作，完成雨污改造三期工程。加快建筑垃圾处置中心建设，推进北区污水处理厂扩建，建成投用餐厨垃圾处置中心、东区污水处理厂。落实“门前三包”责任制，开展生活垃圾强制分类。加强城市公园建设管理，完成东湖南岸公园二期工程，建成环东湖慢道三期、镜台山栈道一期工程。

　　增强县域发展竞争力。坚持沿海与山区联动，开展新一轮山海协同发展行动，促进县域差异化、特色化发展，打造沿海蓝色经济发展带和山区绿色经济增长带。推动沿海县发挥港口优势，引进产业龙头项目，强化产业延链补链，打造具有支撑引领作用的大产业、大集群。引导山区县主动对接四大主导产业，加大招商引资力度，引进落地更多产业链项目。大力发展“飞地”经济，促进上下游配套项目向山区县延伸布局，推动沿海与山区共建产业园区、共享发展效益。深入推动新型城镇化综合试点和“小城市”、小城镇综合改革试点建设，实施省级特色小镇创建项目50个、计划投资40亿元以上。加强乡镇政府服务能力建设，优化基本公共服务资源配置，创新公共服务供给方式。推动农业转移人口城镇落户，实现城镇化率58%以上。

　　（二）坚持铸链育群，打造产业发展新高地

　　做强主导产业。加快补齐产业链条，促进基地化布局、全产业链发展，增强集群发展新优势。围绕加快打造全球规模最大、技术领先的锂电新能源产业基地，加快建设时代新能源三期、新能源科技三期，开工建设杉杉科技二期、屏南新能源三期、思客琦二期，建成投产厦钨二期、屏南新能源二期、时代电机、阿李科技等产业链项目，推进锂电池国家高新技术产业标准化试点建设，力争实现产值600亿元以上。围绕打造东南沿海规模最大、最具竞争力的新能源汽车产业基地，加快上汽宁德基地主机厂和30家配套供应商建设，延伸布局二三级配套供应商，确保6月第一台汽车下线、10月前正式投产，力争全年生产整车4.7万辆。围绕打造全球规模最大、链条完善的不锈钢新材料产业基地，开工建设福安宏泰不锈钢产业科技园，加快建设青拓集团沙湾片区系列不锈钢产业项目、奥展不锈钢产业示范园、柘荣乍洋不锈钢精深加工产业园、荣兴特钢技改项目，力争实现产值1150亿元以上。围绕打造东南沿海规模最大、工艺先进的铜产业基地，推动中铝40万吨铜冶炼项目达产，对接引进铜杆、铜箔等精深加工项目，力争实现产值140亿元以上。推动主导产业融合发展，加快建设青美新能源材料和格林美循环经济产业园。

　　做优传统产业。开展传统产业提档升级行动，实施35项以上重点技改项目。推动电机电器产业加快“机器换工”、智能制造，支持安波、亚南、华益、友力等龙头企业发展超高效节能电机、驱动电机、电喷化油器等中高端机电产品，提升“三电”系统关键零部件研发配套能力。推动合成革产业推广水性、无溶剂、超纤生产工艺，支持邦德、万丰、博艺等龙头企业发展后端精深加工。推动生物医药龙头企业加快开发化学新药、现代中药、生物技术药物，建成广生堂国际认证生产基地、安发生物功能性植物饮料产业化项目。

　　做大现代服务业。推动制造业向研发设计、售后服务等两端延伸，深化“数字宁德”建设，加快布局大数据、云计算、物联网等新兴产业，争取央企和大型民企在宁设立区域总部、研发总部、分拨中心，促进服务业总量扩大、比重提升。坚持“大港口、大物流”，加快市水陆联运中心、国际物流中心建设，深入挖掘漳湾、湾坞、沙埕等港区“散改集”业务潜力，扶持本地陆运航运企业加快成长。坚持“串点连线成面、开发全域旅游”，加快核心景区和旅游小镇建设，加强旅游产品开发创新，加大精准营销和客源市场开拓力度，力争全市游客总人数增长18%、旅游总收入增长27%以上。办好第九届宁德世界地质公园文化旅游节。坚持“商贸业态创新、运营模式创新”，引导龙头企业实施工贸、农贸等主辅分离，力争新增限上贸易企业100家。支持大型电商平台“地方馆”和柘荣、寿宁国家级电子商务进农村综合示范县建设，促进更多名特优产品“上线”卖出去、卖得好。引进培育一批专业市场、商品交易集散地，推进快递电商物流园建设。做好电子商务经营者登记工作。

　　（三）坚持乡村振兴，激活三农发展新动能

　　打赢精准脱贫攻坚战。推动古田、屏南、寿宁三个省级扶贫开发工作重点县和34个建档立卡贫困村有序“摘帽”，确保现行省定标准建档立卡贫困人口全部脱贫。坚持“摘帽”不摘责任、不摘政策、不摘帮扶、不摘监管，加大产业扶贫、就业扶贫、金融扶贫力度，增强造血能力，打牢低水平脱贫人口稳定脱贫基础。落实救助、教育、医疗等帮扶措施，筑牢“减返贫、防新贫”缓冲带。建立4类重点对象危房改造长效机制，完成造福工程搬迁1800人。加强扶贫资金动态监控，确保用在刀刃上、紧要处。加快161个老区基点村发展。实施发展村级集体经济“进五争十”行动，采取资源开发、资产增值、物业租赁等多种方式壮大集体经济。

　　打响现代农业特色牌。全面落实粮食安全责任制，建设水稻生产功能区62.4万亩。坚持规模化、标准化、品牌化、信息化、企业化发展方向，统筹整合涉农资金，加快建设生态茶园、精品果园、放心菜园、营养菌园、道地药园、高山花园和海洋牧场、田园牧场，打造产业兴旺的乡村振兴示范村100个以上，做大做强八大特色农业产业。推动水产湾内标准化设施养殖、湾外深远海养殖、陆上工厂化循环水养殖，建成投用单柱式半潜深海养殖设施，加强福鼎、三沙湾、东冲半岛三个渔港经济区建设。加强茶叶质量安全监管，推行标准化生产，加快打造福鼎白茶、福安红茶两个茶产业集聚中心和蕉城、寿宁、周宁、霞浦、柘荣、古田、屏南七个特色茶产区。大力推广珍稀食用菌、反季节食用菌和代料栽培菌，支持创建国家食用菌产品质量监督检验中心。加强富硒富锌名优特农产品质量体系建设。加快发展高山冷凉花卉产业。引进培育农业产业化龙头企业，提升农产品精深加工水平。推进农村集体产权制度改革，持续开展“两权”抵押贷款试点。推动科技特派员服务向行政村覆盖。

　　打造美丽乡村高颜值。坚持“留白、留绿、留旧、留文、留魂”，提升乡村规划建设水平。开展“一革命四行动”，深化农村人居环境整治。实施农村“厕所革命”，新建改造乡镇公厕48座、农村公厕148座以上，完成户用厕所无害化改造3.5万户。实施农村垃圾治理行动，实现收集转运处理全覆盖。实施农村污水治理行动，新建改造村庄三格化粪池10.87万户，全面建成乡镇生活污水处理设施。实施农房整治行动，推进70%以上村庄房前屋后整治，引导集中建设村镇住宅小区。实施村容村貌提升行动，深化铁路沿线环境整治，推进“三沿一环”绿化提升，改善杭深线宁德段、合福铁路古田段沿线景观面貌。加快“四好农村路”建设，完成农村公路建设改造150公里、安全生命防护工程260公里、危桥改造10座。推动屏南开展农村信息化示范建设。

　　（四）坚持项目带动，强化赶超发展新支撑

　　加强“五个一批”滚动接续。全年计划入库“五个一批”项目1500个，其中谋划200个、签约400个、开工600个、投产255个、增资45个。突出签约、开工、投产三个关键环节，聚焦产业、基础设施、社会民生三大板块，力争项目转化签约率50%、落地率50%、竣工率20%。落实“百千万支撑工程”，实施市在建重点项目300个，完成年度投资618亿元。完善重点项目领导挂钩责任制，坚持计划往前排、服务往前靠、工期往前赶，确保开工90个、竣工90个。

　　加强基础设施领域投资。聚焦交通、能源、水利、电力、通信等领域，加大投资力度，不断提升基础设施供给质量。加快推进温福高铁、宁德至福州长乐机场城际铁路前期工作，争取开工建设漳湾港铁路专用线，全面展开衢宁铁路宁德段施工和上汽铁路专用线建设。扎实推进宁上高速霞浦至福安段、沈海高速宁德段扩容、宁古高速主线工程前期工作，争取早日开工。完成沙埕湾跨海公路通道控制性工程，建成沈海复线福鼎贯岭至管阳段和沈海高速三屿互通、宁古高速六都互通。建成104国道蕉城八都至漳湾疏港公路段拓宽工程。加快推进三都澳深水航道二期工程和漳湾作业区21#、城澳作业区1#2#、8#9#泊位建设，建成沙埕港区进港航道工程和漳湾作业区7#、杨岐作业区16#泊位。力争开工建设宁德核电5#6#机组，加快霞浦核电、周宁抽水蓄能电站建设。新建续建1个500千伏、11个220千伏、26个110千伏输变电工程。开工建设闽清至古田、屏南，福安至周宁、寿宁等天然气支线管网，建成海西天然气二期管网罗源至福鼎段。加快推进赛江防洪三期工程，深化上白石水利枢纽前期工作，开工建设福鼎管阳溪、福安穆阳溪引水工程。完成东湖塘25孔闸除险加固。新建公共停车位1800个、充电桩600个。

　　加强产业项目招商。继续实施千亿招商计划，强化“一把手”招商，“走出去”宣传推介，“请进来”考察对接，加强产业链招商、龙头企业招商，探索推行公司化招商、产业基金招商，精准引进一些投资量大、技术领先、附加值高的大项目，力争工业招商500亿元。深入开展现代服务业招商行动，加强物流、商贸、金融业招商，确保服务业招商400亿元。落实促进开发区高质量发展6条措施，盘活闲置土地、厂房资源，完善基础设施配套，提升园区承载能力，促进一批企业增资扩产，推动产业规模壮大、效益提升。

　　（五）坚持改革开放，增添创新发展新活力

　　突出重点领域改革。深化“放管服”改革，全面推行“证照分离”，推动“多证合一”再升级。完善“双随机一公开”监管机制。加快推进行政审批标准化建设，实现70%以上审批服务事项“一窗受理、集成服务”。推行重大投资项目“模拟审批”，推动工程建设项目审批制度改革，建立以信用监管为核心的建设项目审批事中事后监管新机制。加快实施“互联网+政务服务”，建设全市一体化掌上政务便民服务平台，实现“一网通办、掌上能办”，努力做到“一趟不用跑、最多跑一趟”。深化国有企业改革，调整优化主业范围，健全完善法人治理结构，加快薪酬制度和混合所有制改革，推动国有企业逐步从融资性企业向经营性企业转变。推进建筑产业现代化生产基地建设，鼓励建筑业企业提升资质等级。全面实施预算绩效管理，规范政府举债融资机制，争取地方政府债券50亿元以上。全面推行公立医院人事薪酬制度和医保支付方式改革，完善医疗保障、药品供应制度。落实国家统计制度改革工作，扎实开展第四次全国经济普查。

　　突出加强科技创新。落实加快创新发展和质量强市措施，发挥企业创新主体作用，推动主导产业和新兴产业领域核心技术突破，加强传统产业产品创新。加大财政奖补力度，支持企业建设技术研发平台，联合高校、科研院所共建产业技术创新联盟，力争全市研发投入增长22%以上。新增省级以上高新技术企业20家以上、科技小巨人领军企业10家以上，打造更多单项冠军、隐形冠军和专精特新企业。加快建设电化学储能技术国家工程研究中心、大黄鱼育种国家重点实验室。积极创建能源材料省实验室。加强军民融合深度发展产业园建设，对接引进高新军工企业、科技成果项目，加快“民参军”“军转民”步伐。加强人才队伍建设，加大引进培养力度，支持宁籍人才回归创业。

　　突出营商环境建设。实施营商环境优化提升行动，对标国家、省评价指标体系，全面开展第三方营商环境评估和市对县营商环境指数考核，建立高质高效的经济生态。认真落实促进民营企业加快发展和企业家健康成长的政策措施，放宽民营经济市场准入限制，打造公平竞争的市场环境。推进湾坞-漳湾工业园区增量配电业务改革，支持利用现有工业用地开发建设多层厂房，对行政事业收费能清则清，进一步降低企业合规成本和经营成本，打造适宜企业发展的产业环境。打好金融风险防范化解攻坚战，深化互联网金融、交易场所等领域专项整治，严厉打击非法集资、恶意逃废债行为，力争全市不良贷款率低于全省平均水平、所有县（市、区）控制在2%以下。扶持股权投资类企业发展，对接引进深圳前海等创投基金。加快创建国家普惠金融改革试验区，加大信贷支持力度，力争民营企业年度贷款实际增长10%以上、中小微制造企业贷款增速不低于整体贷款增速水平，打造更加健康诚信的金融环境。落实各项产权保护措施，依法保护企业家人身和财产安全，打造安商护商的法治环境。

　　突出对外开放合作。推动漳湾作业区8#9#10#码头临时开放，争取漳湾作业区正式开放、三沙口岸开放通过国家验收。复制推广自贸试验区、综合实验区政策，加强青拓、中铜、联德等重点企业口岸服务保障，提高口岸监管执法和物流作业效率。积极应对国际经贸形势变化，支持开拓“海丝”沿线国家新兴市场，扩大锂电池、不锈钢、电机电器、水产、食用菌、茶叶等产品出口。深化与台港澳侨交流合作，办好第十届海峡两岸电机电器博览会和第十一届海峡论坛·陈靖姑文化节。

　　（六）坚持铁腕治污，厚植绿水青山新优势

　　深化生态环境建设。坚持开发与保护并重，建立山清水秀的自然生态。落实国家生态文明试验区改革任务，推广应用试点改革成果，加强屏南、周宁、柘荣、寿宁国家重点生态功能区建设。开展生态文明建设年度评价。推进全市“三线一单”编制，启动生态保护红线、永久基本农田、城镇开发边界三条控制线勘界定标、上图入库。加强重点流域生态保护补偿，支持流域范围内县（市、区）环保项目建设。加强矿山、一重山损毁山体植被恢复和三屿园区海洋生态修复。深化国有林场和农垦改革。建设安全生态水系50公里，治理水土流失18万亩。造林绿化4.56万亩，争创国家森林城市。

　　深化污染防治攻坚。加强环保“三合一”督察反馈问题整改，健全环境执法监管新模式，持续打好蓝天、碧水、净土三大保卫战。推动漳湾、湾坞等工业区氮氧化物和挥发性有机物排放削减，加强道路扬尘、餐饮油烟等大气污染防控，坚决遏制臭氧污染。深化河湖长制，加强重点流域及小流域综合整治，完成集中式饮用水水源地环境保护专项治理。深化三都澳海域水环境“1+N”综合整治，认真执行海域养殖“三区”划定方案，严格落实“两个50%”目标，清退渔排10.8万口、藻类1.2万亩，升级改造渔排19.5万口、藻类23.8万亩。加强海漂垃圾治理，推动环三都澳沿岸所有乡镇、村污水处理设施全部达标运营。全面推进土壤污染状况详查，建立土壤环境风险防控体系。

　　深化节能减排工作。完成全市第二次污染源普查。强化能耗、水耗总量和强度“双控”，推动不锈钢新材料、铜材料等产业提质降耗。加快发展节能环保产业，实施节能重点项目10个以上，培育循环经济示范企业10家，推动尾矿、废渣、废旧电子电器等资源综合循环利用。实施排污权有偿使用和交易，开展用能权、碳排放权交易试点，推行合同能源管理、合同节水管理。加强景区、港区、厂区和公共服务领域新能源汽车推广应用，规范共享单车、共享汽车管理。开展“五绿”创建活动，加强工业、建筑、交通、公共机构节能，鼓励绿色低碳生活方式。

　　（七）坚持补齐短板，满足人民群众新期待

　　共织社会保障体系。落实就业优先、支持创业各项政策措施，推行终身职业技能培训制度，新增城镇就业2.4万人、失业人员再就业1万人。加快“全民参保”扩面增缴，推动养老、失业、工伤、基本医疗和生育保险法定人员全覆盖，实现全市城乡低保标准一体化。提高城乡居民医保待遇，市级、市外定点医疗机构和大病保险报销比例分别提高5%，取消大病保险报销封顶线，建档立卡贫困人口市外定点医疗机构报销比例在上述基础上再提高5%。县级医院普通门诊费用纳入医保统筹。做好城镇困难职工解困脱困工作。加强残疾人生活困难救助和发展生产经营项目补助。健全房地产市场健康发展长效机制，编制实施住房发展规划。推进一批棚户区改造项目，改善老城区形象。完成“两违”综合治理370万平方米。支持既有住宅增设电梯。扶持培育住房租赁市场，推行公租房货币化保障，构建租购并举的住房市场和保障体系。

　　共享基本公共服务。建立基本公共服务标准体系，加大民生社会事业补短板力度，年度计划投资42亿元，新建续建项目472个、建成投用项目106个，新增学位1.5万个、医疗床位600张、养老床位1100张以上。加快宁德一中新校区、市医院二期等8个市本级教育、医疗PPP项目建设。新建续建28所公办幼儿园，规范提升一批民办幼儿园。争创义务教育管理标准化学校30所，抓好初中“壮腰”工程，实施高中阶段教育提升计划。加强校外培训机构专项治理，试点推进中小学课后服务。加快发展职业教育，深化产教融合、校企合作，大力培养高素质劳动者和高技能人才。做好宁德师范学院师范类专业二级认证工作。重视发展老年教育、特殊教育和民族教育。支持两大医疗集团做大做强，深化7个县域紧密型医共体内涵建设，加快推进乡村卫生服务一体化。加强产科、儿科等薄弱专科服务能力建设。推动社会福利中心、乡镇敬老院社会化运营，加快金泰园国家级养老标准化试点建设，确保居家养老服务照料中心覆盖所有街道和中心城区乡镇，农村养老服务设施覆盖率达55%，护理型床位占比提高到60%以上。

　　共促文体事业发展。加快建设“四大馆”，开工建设市工人文化宫，深化宁德大剧院、方志馆前期工作。加快县级融媒体中心建设，加强基层综合性文化服务中心资源整合。深入实施“魅力闽东”文化惠民工程，推动《山海的交响》公益展演，支持文艺精品创作。推进闽东苏区红色文化、优秀传统文化保护传承发展。加快省级畲族文化生态保护区和宁德畲族文化园建设。开展第四批23个少数民族特色村寨保护发展试点。深化文化体制改革，加快组建市文化传媒集团，办好畲族歌舞团。支持剪纸、银器等文创产业发展。推动体育场馆社会化、专业化运营，引进品牌体育赛事，深入开展全民健身活动，促进体育、文化、旅游、康养产业融合发展。

　　共建文明和谐家园。开展“创全国文明城 展宁德新形象”全民行动，推进城市管理细化、交通优化、环境净化、市容美化、园林绿化、夜景亮化。加快实施福安新时代文明实践中心全国试点。深入开展扫黑除恶专项斗争，深化社会治安问题专项治理，依法打击盗抢骗、黄赌毒等违法犯罪活动。加大公共安全风险管控力度，深化“雪亮工程”“数字城管”平台应用。完善社区治理，规范物业管理。加强应急管理工作，坚决防范和遏制重特大事故发生。做好防汛防台风等工作，提高防灾减灾救灾能力。加强动植物疫病防控。深化“餐桌污染”治理，推动食品安全“一品一码”全过程追溯体系建设。加强国防动员、后备力量建设，全力做好双拥工作。加强退役军人服务管理。提高依法管理宗教事务水平。发挥工会、共青团、妇联等人民团体作用，做好档案、气象、防震、老龄和关心下一代等工作。

　　三、建设人民满意的服务型政府

　　坚持高质量发展落实赶超，必须以更加优质、高效、规范的政务服务体现新担当、展现新作为。

　　坚定信念，为民服务。深入学习贯彻习近平新时代中国特色社会主义思想和党的十九大精神，树牢“四个意识”，坚定“四个自信”，坚决做到“两个维护”。坚持以人民为中心的发展思想，真诚倾听群众呼声、真情关心群众疾苦，切实为群众多办实事、多解难事。坚持尽力而为、量力而行，办好30件为民办实事项目，将全市一般公共预算支出近八成投入到民生领域，让广大群众有更多、更直接、更实在的获得感。

　　马上就办，勤勉服务。大力弘扬习近平总书记在宁德工作期间留下的好思想好传统好作风，传承“滴水穿石”精神、“弱鸟先飞”意识、“行动至上”作风和“四下基层”制度。推广上汽宁德基地“大干晴天、抢干阴天、巧干雨天、干好每一天”做法，开展重点难点工作专项攻坚，激励广大干部新担当新作为，创造更多的“宁德速度”。坚持政策研究在前、审批服务在前、措施分解落实在前，力戒工作一般性研究、一般性部署、一般性落实，推动机关效能全面提升，为企业和群众提供主动、靠前、细致的服务。

　　依法行政，规范服务。弘扬宪法精神、维护宪法权威，加快建设法治政府，提高政府工作人员法治思维和依法行政能力。扎实推进政府机构改革，加快职能整合、业务融合，严格实施权责清单管理，坚决纠正不作为、慢作为、乱作为现象。依法依规向本级人大及其常委会报告重大行政决策，自觉接受人大法律监督和工作监督、政协民主监督、监察机关监督，重视群众监督、舆论监督，更好发挥审计的重要作用。加强规范性文件备案审核和重大行政决策合法性审查。加强诚信政府建设，健全“政府承诺+社会监督+失信问责”机制，深化政务公开，提升政府公信力。统筹规范督查检查考核，为基层抓落实、抓服务腾出更多时间。

　　从严治党，廉洁服务。以党的政治建设为统领，认真贯彻省委“八个坚定不移”要求，落实意识形态工作主体责任，巩固拓展巡视整改成果，深入推进政府系统党风廉政建设和反腐败工作。持之以恒落实中央八项规定及其实施细则精神和省、市《实施办法》，开展形式主义、官僚主义集中整治。加快构建“亲”“清”政商关系，发挥“政企直通车”平台作用，让服务更暖心、群众更放心、企业家更安心，建立风清气正的政治社会生态。

　　各位代表，新思想引领新时代，新征程呼唤新作为。让我们更加紧密地团结在以习近平同志为核心的党中央周围，在省委、省政府和市委的坚强领导下，不忘初心、牢记使命，团结一心、砥砺奋进，全面实施“一二三”发展战略，为决胜全面建成小康社会、夺取坚持高质量发展落实赶超的新胜利而不懈奋斗！
